# Phenotypic Characteristics and Development of a Hospitalization Prediction Risk Score for Outpatients with Diabetes and COVID-19: The DIABCOVID Study

**DOI:** 10.3390/jcm9113726

**Published:** 2020-11-20

**Authors:** Adèle Lasbleiz, Bertrand Cariou, Patrice Darmon, Astrid Soghomonian, Patricia Ancel, Sandrine Boullu, Marie Houssays, Fanny Romain, Jean Christophe Lagier, Mohamed Boucekine, Noémie Resseguier, Pierre Gourdy, Matthieu Pichelin, Matthieu Wargny, Anne Dutour, Bénédicte Gaborit

**Affiliations:** 1 Department of Endocrinology, Metabolic Diseases and Nutrition, Pôle ENDO, APHM, 13005 Marseille, France; adele.lasbleiz@ap-hm.fr (A.L.); patrice.darmon@ap-hm.fr (P.D.); astrid.soghomonian@ap-hm.fr (A.S.); sandrine.boullu@ap-hm.fr (S.B.); anne.dutour@ap-hm.fr (A.D.); 2 Aix Marseille University, INSERM, INRAE, C2VN, 13005 Marseille, France; patricia.ancel@univ-amu.fr; 3 L’institut du Thorax, Inserm, CNRS, UNIV Nantes, CHU Nantes, Département d’Endocrinologie, Diabétologie et Nutrition, Hôpital Guillaume et René Laennec, 44093 Nantes, France; bertrand.cariou@univ-nantes.fr (B.C.); matthieu.pichelin@chu-nantes.fr (M.P.); 4 Assistance-Publique Hôpitaux de Marseille, Medical Evaluation Department, CIC-CPCET, 13005 Marseille, France; marie.houssays@ap-hm.fr; 5 Public Health and Medical Information Department, APHM, 13005 Marseille, France; fanny.romain@ap-hm.fr; 6 Aix Marseille University, IRD, AP-HM, MEPHI, IHU Méditerranée Infection, 13005 Marseille, France; jean-christophe.lagier@ap-hm.fr; 7 Aix-Marseille University, EA 3279 CEReSS—Health Service Research and Quality of Life Center, 13005 Marseille, France; mohamed.boucekine@univ-amu.fr (M.B.); noemie.resseguier@univ-amu.fr (N.R.); 8 Support Unit for Clinical Research and Economic Evaluation, Assistance Publique-Hôpitaux de Marseille, 13005 Marseille, France; 9 Département d’Endocrinologie, Diabétologie et Nutrition, CHU Toulouse, Institut des Maladies Métaboliques et Cardiovasculaires, UMR1048 Inserm/UPS, Université de Toulouse, 31432 Toulouse, France; pierre.gourdy@inserm.fr; 10 CIC-EC 1413, Clinique des Données, CHU de Nantes, 44000 Nantes, France; matthieu.wargny@chu-nantes.fr

**Keywords:** COVID-19, diabetes, outpatients, hospitalization risk score, DIABSCORE

## Abstract

Diabetes mellitus (DM) has been identified as a risk factor for severe COVID-19. DM is highly prevalent in the general population. Defining strategies to reduce the health care system burden and the late arrival of some patients thus seems crucial. The study aim was to compare phenotypic characteristics between in and outpatients with diabetes and infected by COVID-19, and to build an easy-to-use hospitalization prediction risk score. This was a retrospective observational study. Patients with DM and laboratory- or CT-confirmed COVID-19, who did (*n* = 185) and did not (*n* = 159) require hospitalization between 10 March and 10 April 2020, were compared. Data on diabetes duration, treatments, glycemic control, complications, anthropometrics and peripheral oxygen saturation (SpO_2_) were collected from medical records. Stepwise multivariate logistic regressions and ROC analyses were performed to build the DIAB score, a score using no more than five easy-to-collect clinical parameters predicting the risk of hospitalization. The DIAB score was then validated in two external cohorts (*n* = 132 and *n* = 2036). Hospitalized patients were older (68.0 ± 12.6 vs. 55.2 ± 12.6 years, *p* < 0.001), with more class III obesity (BMI ≥ 40 kg/m^2^, 9.7 vs. 3.5%, *p* = 0.03), hypertension (81.6 vs. 44.3%, *p* < 0.0001), insulin therapy (37% vs. 23.7%, *p* = 0.009), and lower SpO_2_ (91.6 vs. 97.3%, *p* < 0.0001) than outpatients. Type 2 DM (T2D) was found in 94% of all patients, with 10 times more type 1 DM in the outpatient group (11.3 vs. 1.1%, *p* < 0.0001). A DIAB score > 27 points predicted hospitalization (sensitivity 77.7%, specificity 89.2%, AUC = 0.895), and death within 28 days. Its performance was validated in the two external cohorts. Outpatients with diabetes were found to be younger, with fewer diabetic complications and less severe obesity than inpatients. DIAB score is an easy-to-use score integrating five variables to help clinicians better manage patients with DM and avert the saturation of emergency care units.

## 1. Introduction

The epidemic of coronavirus disease 2019 (COVID-19), caused by severe acute respiratory syndrome coronavirus 2 (SARS-CoV-2), reported in Wuhan, China in December 2019, before rapidly spreading worldwide. Pandemic status was declared by the World Health Organization in March 2020 [1]. It surprised many countries with its high propensity rate and broad clinical spectrum. Identifying the people at the greatest risk of developing critical acute respiratory syndrome has become a worldwide challenge. Males, persons aged over 65, and obese individuals are at greater risk of poor prognosis [2]. Besides other underlying diseases such as chronic respiratory disease, cardiovascular disease (CVD), hypertension or cancer, diabetes has been identified as a major comorbidity for illness severity [2,3]. In a comprehensive meta-analysis of 6452 patients, diabetes was associated with mortality, severe COVID-19, acute respiratory distress syndrome and disease progression in patients with COVID-19 [4]. Potential mechanisms that may increase susceptibility to severe COVID-19 in patients with diabetes include higher affinity cellular binding and efficient virus entry, decreased viral clearance, impaired T-cell function, and blunted anti-viral interferon (IFN) responses. Delayed activation of Th1/Th17 increased susceptibility to inflammation and cytokine storm syndrome, as well as CVD. SARS-CoV-2 may also directly disrupt pancreatic beta-cell function through an interaction with ACE2 (angiotensin-converting enzyme 2) [5]. However, whether all individuals with diabetes share the same risk remains to be elucidated [2]. Worldwide, emergency care units have been quickly saturated, and pragmatic risk stratification strategies to decide which patients needed hospitalization have been lacking. Likewise, the routinely available factors to be considered by general practitioners to make clinical decisions on whether to hospitalize or not remain uncertain. Defining a simple risk stratification strategy would be valuable for high-risk patients to mitigate the burden on the healthcare system, to reduce emergency room overcrowding, and to avoid the late arrival of some patients. Furthermore, acute respiratory failure has been shown to be very rapid and brutal, with a dissociation between the severity of hypoxemia and the maintenance of a relatively good clinical presentation before acute respiratory distress syndrome (ARDS) occurs [6,7]. There is a two- to threefold higher prevalence of diabetes in patients in intensive care units (ICUs) compared with those with less severe disease. In the Coronavirus SARS-CoV-2 and Diabetes Outcomes (CORONADO) study, a French multicenter observational study, we previously reported that 29% of 1317 patients with diabetes hospitalized for COVID-19 required intubation or died within 7 days (D7) after admission. Death within D7 was independently associated with older age, treated obstructive sleep apnea syndrome and micro- or macrovascular complications [8]. However, whether this excess mortality was related to severity of COVID-19 *per se* or to comorbid conditions needs more evaluation. In Marseille, the screening strategy for COVID-19 was broad, untargeted, and included early case isolation. In the DIABCOVID study, hospitalized in-patients with diabetes and COVID-19 were compared to outpatients with diabetes and COVID-19 diagnoses screened at the Marseille University Hospital Institute (IHU) during the same period (10 March–10 April), but who did not require hospitalization. The aim of the study was to identify factors associated with hospitalization risk in COVID-19 patients with diabetes, to delineate an upstream care management strategy. We also set out to devise an easy-to-use pragmatic clinical score that would help physicians and general practitioners in deciding the hospitalization of COVID-19 patients with diabetes.

## 2. Methods

### 2.1. Study Design

This was a monocentric study conducted in the 4 Marseille Public Hospitals (APHM) between 10 March and 10 April 2020 during the peak of the epidemic in France. COVID-19 and diabetes diagnosis were retrieved from the medical information department. The conformity of the study with European data privacy rules was approved by the local data protection officer (RGPD PADS20-195) and complied with the Declaration of Helsinki.

Inclusion criteria were COVID-19 diagnosis confirmed biologically (by SARS-CoV-2 PCR test) and/or radiologically (ground-glass opacity and/or crazy paving on chest computed tomography scan) and a personal history of diabetes or newly diagnosed diabetes on admission (glycosylated hemoglobin HbA1c ≥ 6.5% during hospitalization). Hospitalized patients managed at APHM came from the CORONADO study (NCT043224736). Outpatient data came from patients who went to APHM for COVID-19 screening (at IHU) and were positive and managed as outpatients (i.e., did not require hospitalization). Patients were followed up from the day of COVID-19 diagnosis to the day of their release or Day 28 whichever came first. The decision to hospitalize a patient or not was physician-centered and not yet restricted by healthcare resources.

### 2.2. Data Collection

The medical files of inpatients were extracted from the CORONADO study (NCT043224736). The medical files of patients with a history of a diabetes diagnostic code with COVID-19 and who were followed up as outpatients in APHM were extensively reviewed by clinical research associates and physicians. Data were collected from patient’s medical files. Any missing data were exhaustively searched for by contacting general and/or specialist practitioners, regular pharmacists, or biomedical laboratories. The collected data included demographic data (age, sex, ethnicity, autonomy), clinical data (blood pressure, BMI, peripheral oxygen saturation) and information on diabetes such as type of diabetes, duration of diabetes, recent glycemic control (HbA1c determined in the 7 days following admission, or if not available, HbA1c measured in the previous 6 months), severe hypoglycemia, microvascular and macrovascular complications, comorbidities and treatments. Patients were considered autonomous if they lived at home without assistance. Severe hypoglycemia was defined as hypoglycemia requiring the intervention of a third person in the previous year. Microvascular complications were defined as severe diabetic retinopathy (proliferative retinopathy and/or laser photocoagulation and/or clinically significant macular oedema requiring laser and/or intra-vitreal injections) and/or diabetic kidney disease (proteinuria (AER ≥ 300 mg/24 h, urinary albumin/creatinine ratio ≥300 mg/g, urinary albumin/creatinine ratio ≥30 mg/mmol creatinine, proteinuria ≥500 mg/24 h) and/or estimated glomerular filtration rate (eGFR) equal to or lower than 60 mL/min/1.73 m^−2^, using the Chronic Kidney Disease Epidemiology Collaboration (CKD-EPI) formula on the first day of admission) and/or history of diabetic foot ulcer. Macrovascular complications were defined as ischemic heart disease and/or cerebrovascular disease (stroke and/or transient ischemic attack) and/or peripheral artery disease (amputation owing to ischemic disease and/or lower limb artery revascularization). The comorbidities especially searched for were immunodepression (HIV, transplant, active cancer), respiratory (chronic obstructive pulmonary disease, sleep apnea, respiratory failure) or cardiac disease (heart failure, coronary artery disease), and metabolic disease (hepatopathy, bariatric surgery). We also collected COVID-19 symptoms, and radiological and biological values during the first examination. Data on tracheal intubation for mechanical ventilation and death within 28 days of first admission were collected.

### 2.3. Aim of the Study

The aim of our study was to compare the phenotypical characteristics of in- and outpatients with diabetes followed up in APHM for COVID-19 in order to build a risk stratification clinical score to help decide whether to hospitalize patients with diabetes infected by SARS-CoV-2.

### 2.4. Endpoints

The primary endpoint was in- versus outpatient phenotypical status. The secondary endpoints were tracheal intubation for mechanical ventilation and death within 28 days of admission.

### 2.5. Statistical Analysis

Continuous variables were expressed as mean ± standard deviation or median (25th–75th percentile) and were compared using Student’s *t*-test or the Mann–Whitney test. Categorical variables were expressed as frequency and percentage, and were compared using the chi-squared test or Fisher’s exact test. Multivariate logistic regression models were then used to identify independent prognostic factors for hospitalization and to find a final model with readily available variables associated with hospitalization. Stepwise multivariate analysis was then conducted with characteristics prior to admission or at first examination. They were built step-by-step by adding or removing variables based on the results of previous models and the ease in obtaining these variables in general practice. Significant risk factors were assigned weighted points that were proportional to their beta regression coefficient values. Final variables were chosen depending on the best area under the curve (AUC). The variables used in the multivariate analysis had *p*-values < 0.05 in the univariate analysis.

#### 2.5.1. Development of a Risk–Score Model

The variables in the final model were used in the development of the hospitalization risk score. The aim of this analysis was to construct a clinical score that could be easily used in ambulatory medicine and general medicine. Biological and radiological variables were therefore excluded from this analysis. Continuous variables were converted into categorical variables, and cut-off values were determined using ROC curves and the Youden index. The weight of each score variable was then calculated using the formula β-coefficient of the variable in the multivariate analysis model divided by the β-coefficient minimum multiplied by 2 [9]. The reference group of categorical variables was assigned 0 points, corresponding to a β-coefficient of zero.

Receiver operating characteristic (ROC) curve analyses were performed to assess the effectiveness of our risk score for predicting hospitalization in patients with diabetes affected by COVID-19. The cut-off for hospitalization prediction risk score was also determined using ROC curves.

We conducted an internal validation of our score in the cohort with sensitivity, specificity, negative predictive value, and positive predictive value.

In all analyses, two-tailed *p* values < 0.05 were considered to indicate significance. All analyses were performed with available data using SPSS software ver. 20.0 (IBM, Armonk, NY, USA) and the logistf package in R, version 3.6.2 [10].

#### 2.5.2. External Validation of the Risk–Score Model

In order to validate the risk–score model in other populations with diabetes and in another time period of the pandemic, we further tested the diabscore in two other populations with diabetes (*n* = 132 and *n* = 2036)

The first cohort was a cohort of 132 patients with diabetes and COVID-19 hospitalized in summer in APHM when the incidence of COVID-19 was reduced. We chose the period from August the 20th to September the 20th, just before the second wave and the beginning of overnight curfew (RGPD PADS20-195). The 5 clinical data sets used to calculate the diabscore were retrieved from medical records.

The second cohort was the whole CORONADO cohort, a nationwide multicenter observational study in people with diabetes hospitalized for COVID-19 in 68 French centers. If the peripheral saturation of oxygen was not available in ambient air, and the patient was already on oxygen supply on admission, we considered their SpO_2_ to be under 95%.

## 3. Results

### 3.1. Population

**Figure 1:**
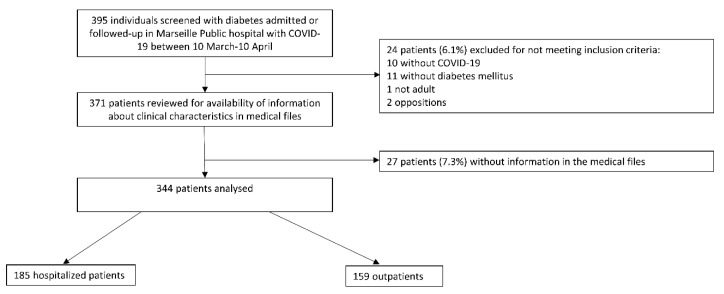
Study flow chart.

In total, 344 patients with diabetes and confirmed COVID-19 referred to Marseille Public Hospitals (APHM) were included—185 patients as patients hospitalized between 10 March and 10 April 2020 for COVID-19, and 159 outpatients followed on an ambulatory basis (Figure 1). Prior to first admission, at least 60% of patients had been followed only by their general practitioner for their diabetes; 20% of hospitalized patients required tracheal intubation, and 13% died within 28 days.

### 3.2. Differences in Demographic and Diabetes-Related Characteristics between Hospitalized and Outpatients

The clinical characteristics of patients are given in Table 1. The completeness of data exceeded 84% except for HbA1c (67%) (Table 1). Fifty-nine percent of the cohort were men, with no significant difference between groups (*p* = 0.27). The study population had a mean age of 62.1 ± 14.0 years, 10.8 ± 8.8 years of diabetes duration and a mean HbA1c of 7.7 (61 mmol/mol) ± 1.7%. As expected, hospitalized patients were older (mean age 68.0 ± 12.6 vs. 55.2 ± 12.6 years, *p* < 0.001), less autonomous (*p* < 0.0001), and more frequently displayed class III obesity (BMI ≥ 40 kg/m^2^, 9.7% vs. 3.5%, *p* = 0.03) than outpatients. Ninety four percent of the study population had type 2 diabetes, with 10 times more type 1 diabetes in the outpatient group (11.3% vs. 1.1%, *p* < 0.0001) (Table 1).

Mean HbA1c value did not significantly differ between groups (7.8 (62 mmol/mol) ± 1.7% inpatients vs. 7.5 (58 mmol/mol) ± 1.6% outpatients, *p* = 0.10). Hospitalized patients had a longer diabetes duration (12.4 ± 9.2 vs. 9.1 ± 8 years, *p* = 0.002), experienced more episodes of severe hypoglycemia in the previous year (*p* = 0.006), and had more microvascular (*p* < 0.0001) and macrovascular (*p* < 0.0001) complications than outpatients. They were also more frequently treated with insulin (37% vs. 23.7%, *p* = 0.009). Comorbidities, especially hypertension (81.6% vs. 44.3%, *p* < 0.0001), dyslipidemia (49.2% vs. 30.8%, *p* = 0.001), obstructive sleep apnea (11.5% vs. 6.2%, *p* = 0.008), respiratory failure (9.8% vs. 1.9%, *p* = 0.003), COPD (7.6% vs. 1.3%, *p* = 0.005), active cancer (6.0% vs. 0.6%, *p* = 0.007), heart failure (9.4% vs. 3.1%, *p* = 0.03) and dialysis (4.3% vs. 0%, *p* =0.008), were more prevalent in hospitalized patients than in outpatients. Inpatients were more frequently being treated with antiplatelet agents (39.7% vs. 12.1%, *p* < 0.0001), renin–angiotensin–aldosterone system (RAAS) blockers (64.7% vs. 33.8%, *p* < 0.0001), diuretics (loop and thiazide, 28.8% vs. 14.7%, *p* = 0.003) and statins (40.8% vs. 25.5%, *p* = 0.004) than outpatients.

### 3.3. Characteristics of COVID-19 during First Examination, and Differences between In- and Outpatients

The characteristics of COVID-19 patients during the first clinical examination are given in Table 2. The mean duration of symptoms before consultation or hospitalization was 6 days in the whole cohort. Outpatients were consulted earlier than hospitalized patients (5.8 vs. 6.2 days, respectively, *p* = 0.002).

Symptoms were present in 94.5%. The most common signs were cough (63.8%), fatigue (60.3%), fever (48.5%) and dyspnea (38.8%), and these were more prevalent in hospitalized patients than in outpatients (all *p* < 0.05). By contrast, cephalalgia, anosmia and/or ageusia and pharyngeal symptoms were more prevalent in out- than in inpatients (all *p* < 0.0001). SARS-CoV-2 PCR testing was performed in all the patients, with a positive result in 96%. In 14 patients for whom the PCR was negative but with typical pulmonary CT findings, the diagnosis of COVID-19 was rectified. Thoracic CT imaging, available for 288 patients (84%), demonstrated typical COVID-19 pneumopathy signs (ground-glass opacity and/or crazy paving) in 85.4%; severe forms predominated in hospitalized patients (32.1% vs. 2% respectively, *p* < 0.05) compared to outpatients.

Ketosis was present in 2.1% of patients on admission and did not significantly differ between groups. Ambient air peripheral oxygen saturation at first examination was lower in hospitalized patients (91.6% vs. 97.3%, *p* < 0.0001) than in outpatients.

Numerous biological variables differed between in- and outpatients. Hospitalized patients had lower hemoglobin (13.1 ± 2.0 vs. 13.8 ± 1.5 g/dL, *p* < 0.0001), lymphocyte counts (1.2 ± 0.9 vs. 1.8 ± 0.8 G/L, *p* < 0.0001), platelet counts (211 ± 84 vs. 244 ± 83 103/mm^3^, *p* < 0.0001) and albumin (37 ± 5 vs. 43 ± 4 g/L, *p* < 0.0001) than outpatients. Conversely, inpatients displayed more inflammation (CRP 88.6 ± 81.8 vs. 18.9 ± 33.6 mg/L, *p* < 0.0001), higher neutrophil counts (5.1 ± 2.8 vs. 3.6 ± 1.5 G/L, *p* < 0.0001), higher admission plasma glucose levels (10.7 ± 5.8 vs. 9.2 ± 4.2 mmol/L, *p* = 0.01) and higher values for cellular lysis, i.e., ASAT (50 ± 35 vs. 34 ± 18, *p* < 0.0001), CPK (314 ± 831 vs. 104 ± 65, *p* = 0.001) and LDH (351 ± 155 vs. 233 ± 56, *p* < 0.0001), than outpatients (Table 2).

### 3.4. Predictive Criteria for Hospitalization and Model Development of an Easy-to-Use Pragmatic Score for Clinicians

Many variables were linked to the risk of hospitalization in univariate analysis, and multiple logistic regression models were performed to find factors independently associated with the hospitalization risk.

The different models are presented in Table 3.

Age, hypertension, BMI and plasma glucose at admission, peripheral oxygen saturation, micro- and macroangiopathy, and insulin treatment were independently associated with hospitalization risk (*p* < 0.05). Type of diabetes did not systematically remain associated with hospitalization risk depending on covariates, because of the interaction between variables, and the odds ratio remained high but with a large confidence interval (Table 3).

**Figure 2:**
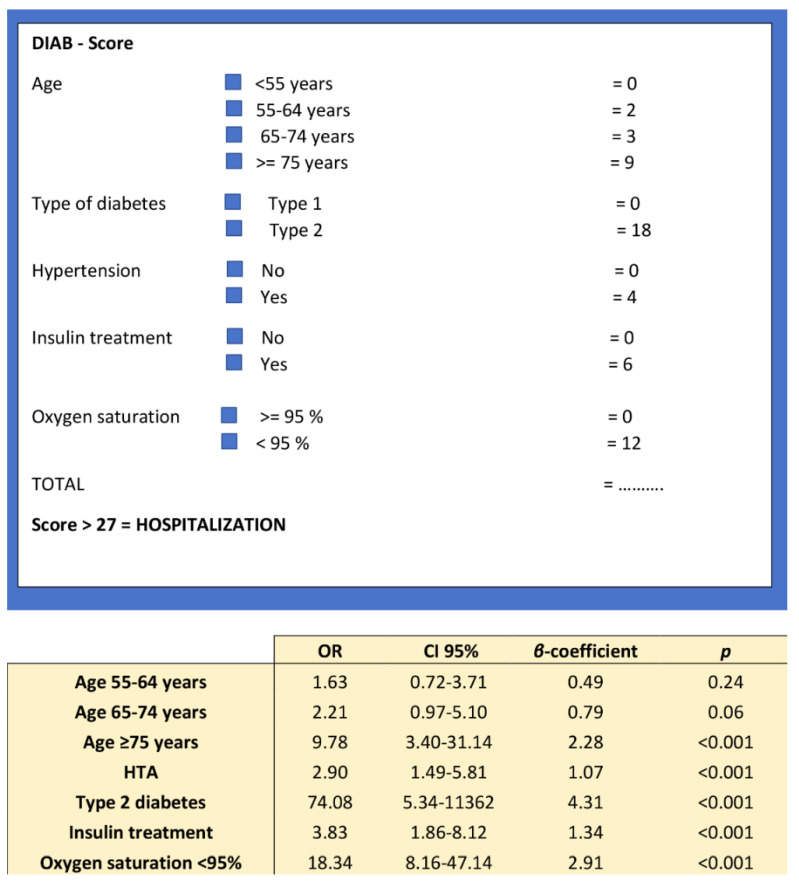
Diabscore: an easy-to-use hospitalization risk score.

Model 7 provides the highestAUC, and was chosen to build the diabscore, an easy-to-use pragmatic prognostic score for hospitalization (Figure 2).

The final score included different age categories (≥75 years old, OR = 9.8; 95% CI 3.4–31.1), type of diabetes (OR = 74.1; 95% CI 5.3–11362), presence of hypertension (OR = 2.9; 95% CI 1.5–5.8), insulin treatment (OR = 3.8; 95% CI 1.9–8.1) and peripheral oxygen saturation (OR = 18.3 95% CI 8.2–47.1). The best cut-off value for peripheral oxygen saturation was identified by the Youden index (Youden = 0.55) at 95%.

**Figure 3:**
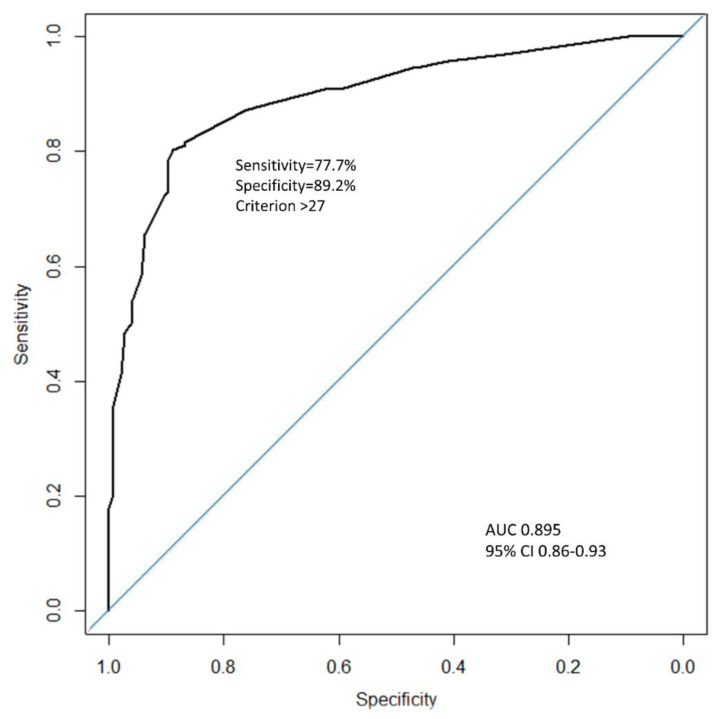
ROC curve of the DIABCOVID hospitalization predicting score (black curve) and a random classifier (blue curve).

The maximum allocated score was 49 and the model was developed to be calculated if at least four of the five variables were available. There were 39 missing data (31 concerned oxygen saturation). The cut-off value to predict hospitalization was 27 (Youden = 0.67). A score above 27 was strongly associated with hospitalization (OR = 28.9; 95% CI 15.7–53.3). A diabscore > 27, AUC = 0.895, had a sensitivity (Se) of 77.7%, a specificity (Sp) of 89.2%, a positive predictive value of 89.4%, and a negative predictive value of 77.5% (Figure 3).

### 3.5. Secondary Endpoints

The secondary endpoints were tracheal intubation for mechanical ventilation and death within 28 days of admission. A diabscore >27 predicted tracheal intubation (Se = 81.6%, S*p* = 57.6%) and death (Se = 96%, S*p* = 57.1%) by day 28.

A receiver operating characteristics (ROC) curve was used to identify the best cut-off value for the prediction of hospitalization in COVID-19 patients with diabetes. AUC, area under the curve.

### 3.6. External Validation of the Diabscore in Two Other Cohorts

#### 3.6.1. External Validation of the Diabscore in Another Period of Time

We first evaluated our score later in the summer of 2020 when the incidence of COVID-19 was reduced. The main characteristics of the cohort of patients with diabetes and COVID-19 hospitalized in summer in APHM are shown in Supplementary Table S1. In this cohort, 78.0% of the population had a score above 27 (95% CI according to Clopper–Pearson estimate: 70.0–84.8%).

#### 3.6.2. External Validation of the Diabscore in the Whole CORONADO Cohort

We then tested our score on the whole nationwide multicentric CORONADO cohort. Among the 2842 patients with diabetes and included in the CORONADO trial, 2036 patients had available data to calculate the diabscore. A diabscore >27 was found in 1722/2036 patients, i.e., 84.6% of the study population (95% CI according to Clopper–Pearson estimate 82.9–86.1%). Death within 28 days occured in 6.1% of patients with a diabscore ≤ 27, whereas it reached 22.5% of patients with a diabscore > 27, *p* < 0.0001.

## 4. Discussion

DIABCOVID is one of the first studies dedicated to outpatients with diabetes infected by SARS-CoV-2. It was designed to compare the phenotypic differences of outpatients with diabetes and hospitalized patients with COVID-19 diagnosis, and aimed at identifying predictive factors for hospitalization in this specific population, known to be at high risk for severity. Considering variables prior to first examination in our cohort, hospitalized patients were older, had more massive obesity, type 2 diabetes with a longer duration and more macro- and microvascular complications together with comorbidities and treatment with insulin. An American retrospective study analyzed disease outcomes for 49 patients with diabetes between 16 March and 24 April 2020, with similar age and HbA1c but higher BMI than our cohort, who were managed as outpatients and followed via audiovisual telemedicine [11]. Interestingly, the authors observed that the rate of hospitalization for patients with diabetes was double the rate for their patients overall. However, specific diabetes characteristics leading to hospitalization were not analyzed.

In the DIABCOVID study, remarkably severe hypoglycemia and long-term glycemic control were not independently associated with hospitalization as opposed to plasma glucose level at admission. The number of patients with ketosis was not significantly different between in- and outpatients (*p* = NS), suggesting that neither acute diabetic complications nor poor preexisting glycemic control on admission drove the hospitalization decision. An increased plasma glucose level at admission may reflect stress-induced hyperglycemia and could reflect the severity of SARS-CoV-2 infection, or new onset diabetes, whose incidence was found to be increased during the COVID-19 outbreak [12]. These results are consistent with those of the CORONADO study or the study of Zhu et al., who showed that an increased plasma glucose level was significantly associated with worse outcomes in patients with diabetes [8,13].

The DIABCOVID score was built as an easy-to-use score, with only five components that are easy to obtain during a general practice consultation for individuals with diabetes, and that were independently associated with the risk of hospitalization. This score used one demographical variable (age), three variables of medical history (type of diabetes, insulin treatment, hypertension) and one clinical variable (peripheral oxygen saturation on room air at admission). It predicted hospitalization with an AUC of 0.895.

Age above 75 years and hypertension were independently associated with hospitalization (OR of 9.8 and 2.9 respectively) in our model. This is in line with all previous epidemiological studies showing that older patients are at greater risk in terms of severity, and hypertension was found to be the most common comorbidity present in hospitalized patients [14,15,16,17]. Accordingly, we found that ACE inhibitors and/or ARBs or diuretics were more frequently seen among in- than among outpatients. Unsurprisingly, insulin treatment was associated with hospitalization (OR of 3.83), and hospitalized patients very frequently exhibited complications of diabetes (microvascular and macrovascular complications) compared to outpatients. This is in line with the latest recommendations of American Diabetes Association (ADA) and European Association for the Study of Diabetes (EASD) for the management of hyperglycemia in T2D [18], and suggests that hospitalized T2D patients displayed diabetes for a longer duration, or displayed a more advanced stage of their disease or comorbidities that prevented the continuation of other antidiabetic treatments.

We observed that COPD was more prevalent in inpatients compared to outpatients. However, COPD did not remain independently associated with hospitalization risk. Conversely, the CoViDiab I and II studies showed that COPD was significantly associated with a higher risk of both COVID-19 hospitalization and progression towards bad outcomes [19,20].

While it is well known that obesity is associated with the severity of COVID-19 [21], with a French study showing that 76% of patients admitted into the ICU for COVID-19 were at least overweight [22], we showed in our cohort that only BMI > 40 kg/m^2^ was associated with the risk of hospitalization after adjustment for age, sex, insulin treatment and type of diabetes. In a recent systematic review of published data on obesity and COVID-19, a pooled analysis showed that individuals with obesity were more at risk of being COVID-19 positive, 113% more at risk of hospitalization (*p* < 0.0001), 74% more at risk for ICU admission, and 48% more (*p* < 0.001) at risk for mortality [23]. In our cohort, mean BMI was relatively high (29.5 ± 6.6 kg/m^2^) and did not differ between in- and outpatients, which probably accounts for the fact that only massive obesity predicted hospitalization risk.

An interesting finding of the DIABSTUDY was that most of the patients with T1D could be managed in an outpatient setting. It was already known that type 2 diabetes mellitus patients were at high risk of severe forms of COVID-19 [24], and a recent report from the COVID-19-Associated Hospitalization Surveillance Network (COVID-NET) confirmed that hospitalization rates were three times higher in patients with diabetes [25]. However, very few studies have reported COVID-19 outcomes in type 1 diabetes [26]. In our cohort, 5.8% of patients were T1D, 90% were managed as outpatients, and only 10% needed hospitalization. All these T1D patients were younger (mean age 40.1 ± 15 vs. 63.5 ± 13 years) and thinner (mean BMI 25.5 ± 5 vs. 29.7 ± 7 kg/m^2^), but with poorer long-term glucose control (mean HbA1c 8.5 ± 1.7% vs. 7.6 ± 1.7%) and with a longer diabetes duration (mean 14.8 ± 12 vs. 10.5 ± 8 years) than T2D. After adjustment for age and BMI, patients with T2D always had a much greater risk of being hospitalized, with an OR = 74, than T1D. This lower-than-expected prevalence of T1D among patients hospitalized for COVID-19 is consistent with the CORONADO study, which reported a prevalence of 2.1% of T1D hospitalized with COVID-19 [26], and a threefold lower risk of tracheal intubation and/or death by day 7 in T1D <55 years of age. Although these results need to be confirmed in dedicated T1D prospective studies, there seems to be a lower risk of hospitalization in patients with T1D, which could help clinicians triage patients with diabetes in general practice or emergency rooms.

The last component of our easy-to-use score was peripheral oxygen saturation in room air, which was independently associated with hospitalization, with an OR = 18 just after type of diabetes, and which prevailed over BMI in predicting hospitalization risk. It is now well accepted that hypoxia is significantly associated with mortality and severity in patients with COVID-19 [27,28], and cases having no or minimal symptoms, but with markedly reduced pulse oximetry readings, referred to as having “silent” hypoxia, have been shown to be associated with clinical deterioration [7]. In the 4C Mortality Score validated in 22,361 patients admitted to hospital, peripheral oxygen saturation in room air ≥ or <92% discriminately predicted in-hospital mortality [29]. In a retrospective study including 140 patients with moderate to critical COVID-19-associated pneumonia requiring oxygen supplementation, SpO_2_ below 90% was associated with mortality, with a hazard ratio of 47.4 [27]. However, 95% is the threshold defining normal saturation. This is consistent with the French public health authority (Haut Conseil de la santé publique, HSCP, France) recommendation for hospitalization in cases of pneumonia with peripheral oxygen saturation below 95%.

All these factors enabled us to build an easy-to-use score for predicting hospitalization if the score was above 27 with an AUC = 0.895, a good specificity of 89.2% and a sensitivity of 77.7%. A retrospective analysis using the DIAB score in patients of the DIABSTUDY, who had had an ambulatory visit before their hospitalization, showed that 9/13 patients (70%) could have been hospitalized earlier (DIAB score >27). This could have an impact on the triaging of patients with diabetes in an emergency setting, and if healthcare resources are limited.

Moreover, the diabscore was validated in two external cohort patients, one at a different time period of the epidemic (with less cases) and one in the whole multicentric CORONADO cohort that included more than 2000 patients. Despite the fact that these cohorts included only hospitalized patients, this demonstrated the robustness of the diabscore to predict hospitalization, and this was also true for death at day 28.

This study, however, has some limitations. Our cohort was small compared to the current databases on COVID-19. However, to our knowledge, it is so far the largest cohort with outpatients with diabetes. A second limitation is the large proportion of patients without available HbA1c (33%), which may have biased our results. This is nevertheless in line with the observation that only 55% of individuals with diabetes have had three or more HbA1c determinations in the previous year according to French national registry data [30]. Another limitation is the absence of any external validation of the score in an ambulatory setting, due to the scarcity of outpatient studies on COVID-19. Prediction models for the diagnosis and prognosis of COVID-19 have been evaluated in a recent systematic review and show a high risk of bias and probably optimistic performances [31]. Given this crucial limitation, we are open to sharing individual participant data to validate and perhaps build a new, more rigorous score. In our study, there was a majority of T2D, which could have biased our score involving the type of diabetes. Finally, the hospitalization of patients may have depended on the degree of saturation of the health system, and some patients who would have been hospitalized in another context may not have been because of an overload of reception capacities at this time. An Italian study showed that during the COVID-19 outbreak, calls for out-of-hospital emergency systems strongly increased (up to +440%) along with attendance in emergency care units [32]. However, the diabscore performed well in another period of the pandemic with a lower incidence of COVID-19 cases, highlighting its robustness for predicting hospitalization risk. Therefore, the major strength of this study is the access to outpatients, enabling us to identify the patients with diabetes at risk of severe forms of COVID-19 requiring hospitalization, but also those who can be reassured.

In conclusion, the DIABCOVID study uniquely reports the phenotypes of COVID-19 individuals with diabetes admitted to hospital or followed up in an ambulatory setting. Advanced age, insulin treatment, type 2 diabetes, presence of hypertension and low peripheral saturation were strongly associated with risk of hospitalization, and might require specific management. By contrast, patients with type 1 diabetes were less at risk of being hospitalized and could be reassured. All these findings could guide clinicians to best advise their patients and avoid a loss of chance, or prevent the overload of emergency care units.

## Figures and Tables

**Table 1:** Clinical characteristics prior to first examination.

Variable	Data Available	All (*n* = 344)	Outpatients (*n* = 159)	Inpatients (*n* = 185)	*p*
Sex male/female	344	204/140 (59.3%)	89/70 (56%)	115/70 (62.1%)	NS
Age (years)	344	62.1 ± 14.0	55.2 ± 12.6	68 ± 12.6	<0.0001
<55 years		100 (29.1%)	77 (48.4%)	23 (12.4%)	<0.0001
55–64 years		94 (27.3%)	46 (28.9%)	48 (25.9%)	ref
65–74 years		82 (23.8%)	29 (18.2%)	53 (28.6%)	0.07
≥75 years		68 (19.8%)	7 (4.4%)	61 (33%)	<0.0001
BMI (kg/m^2^)	308	29.5 ± 6.6	29 ± 5.3	29.8 ± 7.5	NS
<25 kg/m^2^		72 (23.4%)	31 (21.7%)	41 (24.8%)	NS
25–29.9 kg/m^2^		114 (37%)	58 (40.6%)	56 (33.9%)	NS
30–39.9 kg/m^2^		101 (32.8%)	49 (34.3%)	52 (31.5%)	NS
≥40 kg/m^2^		21 (6.8%)	5 (3.5%)	16 (9.7%)	0.030
Autonomy	344				<0.0001
Autonomous		307 (89.2%)	159 (100%)	148 (80%)	
Non-autonomous		37 (10.8%)	0 (0%)	37 (20%)	
Ethnicity	292				0.010
EU		103 (35.3%)	43 (28.3%)	60 (42.9%)	ref
MENA		123 (42.1%)	75 (49.3%)	48 (34.3%)	0.004
AC		64 (21.9%)	34 (22.4%)	30 (21.4%)	0.153
AS		2 (0.7%)	0 (0%)	2 (0.7%)	NA
Hypertension	343	221 (64.4%)	70 (44.3%)	151 (81.6%)	<0.0001
Dyslipidemia	342	139 (40.6%)	49 (30.8%)	90 (49.2%)	0.001
Tobacco use	320				<0.0001
Never		236 (73.8%)	136 (85.5%)	100 (62.1%)	
Former		56 (17.5%)	6 (3.8%)	50 (31.1%)	
Current		28 (8.8%)	17 (10.7%)	11 (6.8%)	
Type of diabetes	344				<0.0001
Type 1		20 (5.8%)	18 (11.3%)	2 (1.1%)	
Type 2		324 (94.2%)	141 (88.7%)	183 (98.9%)	
Diabetes duration	267	10.8 ± 8.8	9.1 ± 8	12.4 ± 9.2	0.002
HbA1c (%)	229	7.7 ± 1.7	7.5 ± 1.6	7.8 ± 1.7	0.100
Severe hypoglycemia	285	12 (4.2%)	1 (0.7%)	12 (4.2%)	0.006
Microvascular complications	315	109 (34.6%)	23 (16.7%)	86 (48.6%)	<0.0001
Severe diabetic retinopathy	294	14 (4.7%)	6 (4.7%)	8 (4.8%)	NS
Diabetic kidney disease	344	104 (30.2%)	21 (13.2%)	83 (44.9%)	<0.0001
History of diabetic foot ulcer	344	6 (1.7%)	2 (1.3%)	4 (2.2%)	NS
Macrovascular complications	342	71 (20.8%)	14 (8.9%)	57 (31%)	<0.0001
Ischemic heart disease	342	58 (17%)	10 (6.3%)	48 (26.2%)	<0.0001
Cerebrovascular disease	338	21 (6.1%)	5 (3.2%)	16 (8.7%)	0.040
Peripheral artery disease	340	14 (4.1%)	2 (1.3%)	12 (6.5%)	0.020
Comorbidities					
CKD *	331	49 (13.8%)	1 (0.7%)	48 (26%)	<0.0001
Dialysis	344	8 (2.3%)	0 (0%)	8 (4.3%)	0.010
Heart failure	340	22 (6.5%)	5 (3.1%)	17 (9.4%)	0.030
Sleep apnea	321	37 (11.5%)	9 (6.2%)	37 (11.5%)	0.010
Respiratory failure	342	21 (6.1%)	3 (1.9%)	18 (9.8%)	0.003
COPD	343	16 (4.7%)	2 (1.3%)	14 (7.6%)	0.005
Active cancer	341	12 (3.5%)	1 (0.6%)	11 (6%)	0.007
Transplant	344	4 (1.2%)	1 (0.6%)	3 (1.6%)	NS
NAFLD or liver cirrhosis	337	24 (7%)	12 (7.5%)	12 (6.7%)	NS
Bariatric surgery	344	3 (0.9%)	2 (1.3%)	1 (0.5%)	NS
Treatments					
Insulin	337	104 (30.9%)	37 (23.7%)	67 (37%)	0.010
Basal bolus regimen	333	66 (19.8%)	26 (16.9%)	40 (22.3%)	0.012
Metformin	339	214 (63.1%)	104 (67.1%)	110 (59.8%)	NS
DPP4-Inhibitors	340	79 (23.2%)	37 (23.7%)	42 (22.8%)	NS
GLP1-RA	340	38 (11.2%)	17 (10.9%)	21 (11.4%)	NS
Glinides	339	35 (10.3%)	13 (8.4%)	22 (12%)	NS
Sulfonylurea	339	72 (21.2%)	33 (21.3%)	39 (21.2%)	NS
Anti-platelet agent	341	92 (27%)	19 (12.1%)	73 (39.7%)	<0.0001
ACE inhibitors and/or ARBs	341	172 (50.4%)	53 (33.8%)	119 (64.7%)	<0.0001
Diuretics	340	76 (22.4%)	23 (14.7%)	53 (28.8%)	0.003
Statins	341	115 (33.7%)	40 (25.5%)	75 (40.8%)	0.004
Anticoagulant	341	28 (8.1%)	4 (2.6%)	24 (13%)	0.001

Data are presented as numbers (%) and mean ± SD. NS: non-significant, NA: not applicable, ref: reference. CKD: chronic kidney disease (* defined as eGFR < 45 mL/min/ 1.73 m^2^), COPD: chronic obstructive pulmonary disease. Ethnicity: EU (Europid), MENA (Middle Eastern and North African), AC (African and Caribbean), AS (Asian). HbA1c corresponds to the HbA1c value determined in the 6 months prior to or in the first 7 days following hospital admission. DKD: diabetic kidney disease defined as estimated glomerular filtration rate eGFR ≤ 60 mL/min and/or proteinuria. NAFLD: non-alcoholic fatty liver disease, GLP1-RA: glucagon-like peptide 1-receptor agonist, DPP-4 inhibitors: dipeptidyl peptidase-4, ACE inhibitors: angiotensin-converting enzyme inhibitors, ARBs: angiotensin II receptor blockers.

**Table 2:** COVID-19-related clinical, radiological, and biological characteristics on first examination (at the emergency department unit or IHU).

Variable	Data Available	All (*n* = 344)	Oupatients (*n* = 159)	Inpatients (*n* = 185)	*p*
Positive SARS-CoV-2-PCR	344	330 (96%)	158 (99.4%)	172 (93%)	0.002
Typical CT signs	288	246 (85.4%)	96 (78%)	150 (88%)	
limited		87 (35.3%)	59 (61.5%)	28 (19%)	<0.05
limited to intermediate		4 (1.6%)	0 (0%)	4 (2.6%)	NS
intermediate		96 (39%)	35 (36.5%)	61 (41%)	NS
intermediate to severe		8 (3.2%)	0 (0%)	8 (5.3%)	NS
severe		51 (2.2%)	2 (2%)	49 (32.1%)	<0.05
COVID-19 symptoms	343	324 (94.5%)	144 (90.6%)	180 (97.8%)	0.004
Fever	340	165 (48.5%)	47 (29.6%)	118 (65.2%)	<0.0001
Cough	340	217 (63.8%)	92 (57.9%)	125 (69.1%)	0.040
Dyspnea	343	133 (38.8%)	27 (17%)	106(57.6%)	<0.0001
Cephalalgia	339	77 (22.7%)	51 (32.1%)	26 (14.4%)	<0.0001
Anosmia and/or agueusia	340	115 (33.4%)	85 (53.5%)	30 (16.6%)	<0.0001
Fatigue	340	205 (60.3%)	80 (50.3%)	125 (69.1%)	0.001
Rhinitis and/or pharyngeal symptoms	340	69 (20.3%)	55 (34.6%)	14 (7.7%)	<0.0001
Digestive disorder	340	77 (22.6%)	31 (19.5%)	46 (25.4%)	NS
Time between symptoms and first day hospital or consultations	332	6 ± 4.6	5.8 ± 4.5	6.2 ± 4.6	0.002
Secondary infection	336	19 (5.7%)	0 (0%)	19 (10.7%)	<0.0001
Ketosis	340	7 (2.1%)	2 (1.3%)	5 (2.8%)	NS
Peripheral oxygen saturation (%)	313	94.3 ± 6.7	97.3 ± 1.5	91.6 ± 8.3	<0.0001
Biology at admission					
Hemoglobin (g/dL)	330	13.4 ± 1.8	13.8 ± 1.5	13.1 ± 2.0	<0.0001
White cell count (G/L)	330	6.6 ± 2.7	6.0 ± 1.7	7.0 ± 3.2	<0.0001
Lymphocyte count (G/L)	311	1.5 ± 0.9	1.8 ± 0.8	1.2 ± 0.9	<0.0001
Neutrophil count (G/L)	311	4.4 ± 2.4	3.6 ± 1.5	5.1 ± 2.8	<0.0001
Eosinophil count (G/L)	311	0.02 (0–0.07)	0.07 ± 0.1	0.04 ± 0.144	NS
Platelet count (10^3^/mm^3^)	330	225 ± 85	244 ± 83	211 ± 84	<0.0001
eGFR (/min)	331	77 ± 28.8	91.8 ± 21.7	66.2 ± 28.7	<0.0001
Admission plasma glucose (mmol/L)	324	10 ± 5.2	9.2 ± 4.2	10.7 ± 5.8	0.010
ASAT (UI/L)	292	44 ± 30	34 ± 18	50 ± 35	<0.0001
ALAT (UI/L)	292	38 ± 28	39 ± 26	37 ± 30	NS
GGT (UI/L)	292	44 (26–69)	57 ± 58	73 ± 123	NS
CRP (mg/L)	299	32 (5.8–87)	18.9 ± 33.6	88. ± 81.8	<0.0001
CPK (UI/L)	280	100 (59–191)	77 (56–134)	121 (67–257)	0.001
LDH (UI/L)	269	304 ± 138	232.6 ± 56	351.2 ± 155	<0.0001
Albumin (g/L)	211	38 ± 6	43 ± 4	37 ± 5	<0.0001

Data are represented as numbers (%) and mean ± SD or median (25th–75th percentile). eGFR, estimated glomerular filtration rate eGFR was calculated according to the CKD-EPI formula. GGT, gamma glutamyl transferase. LDH, lactate dehydrogenase.

**Table 3:** Stepwise multivariate analysis.

	AUC	OR	95% CI	β Coefficient	*p*
Model 1—Basic medical history	0.814				
Age		1.07	(1.04–1.10)	0.07	<0.001
Sex		0.71	(0.41–1.23)	−0.34	0.22
Type of diabetes		2.99	(0.58–15.44)	1.10	0.19
Hypertension		2.94	(1.65–5.25)	1.09	<0.001
COPD		5.18	(0.94–28.43)	1.64	0.06
BMI (< or ≥ 40)		3.83	(1.20–12.22)	1.34	0.02
Model 2—Medical history and biological data at first examination	0.860				
Age		1.07	(1.05–1.10)	0.07	<0.001
Sex		0.63	(0.35–1.14)	−0.45	0.13
Type of diabetes (T2D vs T1D)		3.94	(0.68–22.9)	1.37	0.13
Hypertension		3.91	(2.08–7.35)	1.36	<0.001
BMI (< or ≥ 40)		4.39	(1.28–15.01)	1.48	0.02
CKD		28.1	(3.55–222.33)	3.34	0.002
Plasma glucose at admission		1.14	(1.07–1.22)	0.13	<0.001
Model 3—Medical history and long-term plasma glucose	0.830				
Age		1.08	(1.04–1.12)	0.08	<0.001
Sex		0.62	(0.30–1.28)	−0.47	0.2
Type of diabetes		3.83	(0.77–82.54)	2.08	0.08
Hypertension		3.82	(1.79–8.15)	1.34	<0.001
BMI (< or ≥ 40)		5.43	(0.81–36.45)	1.69	0.08
HbA1c		1.02	(0.80–1.29)	0.02	0.88
Plasma glucose at admission		1.12	(1.02–1.24)	0.18	0.02
Model 4—Medical history and antidiabetic treatment characteristics	0.825				
Age		1.07	(1.03–1.09)	0.06	<0.001
Sex		0.67	(0.37–1.21)	−0.41	0.18
Type of diabetes		24.51	(1.95–307.56)	3.20	0.01
Hypertension		2.68	(1.43–5.05)	0.99	0.002
BMI		4.72	(1.26–17.65)	1.55	0.02
Severe hypoglycemia		7.12	(0.55–91.58)	1.96	0.13
Insulin treatment		2.49	(1.20–5.17)	0.91	0.01
Model 5—Medical history and diabetes complications	0.831				
Age		1.05	(1.03–1.08)	0.05	<0.001
Sex		0.83	(0.46–1.49)	−0.19	0.53
Type of diabetes		4.24	(0.78–23)	1.44	0.09
Hypertension		2.29	(1.23–4.28)	0.83	0.01
BMI (< or ≥ 40)		4.07	(1.26–13.14)	1.40	0.02
Microangiopathy		2.11	(1.10–4.05)	0.74	0.02
Macroangiopathy		3.35	(1.50–7.52)	1.21	0.003
Model 6—Medical history and data at first examination	0.910				
Age		1.03	(1.00–1.06)	0.03	0.03
Sex		0.92	(0.46–1.84)	−0.08	0.82
Type of diabetes		45.34	(2.96–7033.84)	3.81	<0.001
Hypertension		3.51	(1.63–7.90)	1.25	<0.001
BMI (< or ≥ 40)		4.14	(0.92–21.17)	1.42	0.06
Peripheral oxygen saturation		0.58	(0.47–0.69)	−0.54	<0.001
Insulin treatment		3.98	(1.82–9.02)	1.38	<0.001
Model 7- Clinical model simplified	0.910				
Age		1.03	(1.01–1.06)	0.03	0.01
Type of diabetes		52.39	(3.42–8067.31)	3.95	<0.001
Hypertension		3.35	(1.66–7)	1.21	<0.001
Peripheral oxygen saturation		0.56	(0.46–0.66)	−0.59	<0.001
Insulin treatment		3.70	(1.75–8.09)	1.31	<0.001

Age presented as continuous variable. CKD, chronic kidney disease.

## Supplementary Materials

The following are available online at https://www.mdpi.com/2077-0383/9/11/3726/s1, Table S1: Main Characteristics of Patients with Diabetes Hospitalized in APHM during Summer 2020 (from 20 August 2020 to 20 September 2020) just before the Recrudescence of the Epidemic Second Wave).
