# Supplementary material for: Phenotypic Characteristics and Development of a Hospitalization Prediction Risk Score for Outpatients with Diabetes and COVID-19: The DIABCOVID Study
Source: J Clin Med. 2020 Nov 20;9(11):3726. doi: 10.3390/jcm9113726 (PMC7699790; doi:10.3390/jcm9113726)
Supplement: Supplementary file 1 [file jcm-09-03726-s001.pdf]

Supplementary table 1:

Table 1. Main Characteristics of Patients with Diabetes Hospitalized in APHM during Summer 2020 (from 20th of August to 20th of September) just before the Recrudescence of the Epidemic Second Wave).

Age (years)	69 [62 – 81]
- < 55 (n, %)	18 (13.6)
- 55-64 (n, %)	23 (17.4)
- 65-74 (n, %)	40 (30.3)
- ≥ 75 (n, %)	51 (38.6)
Sex (n, % M)	80 (60.1)
Type 2 Diabetes (n, %)	128 (97.0)
Hypertension (n, %)	88 (66.7)
Insulin treatment (n, %)	32 (24.2)
Oxygen Saturation (n, %)	
- ≥ 95%	43 (32.6)
- < 95%	89 (67.4)
